# Multiple Sensor Monitoring of CFRP Drilling to Define Cutting Parameters Sensitivity on Surface Roughness, Cylindricity and Diameter

**DOI:** 10.3390/ma13122796

**Published:** 2020-06-21

**Authors:** Miguel Álvarez-Alcón, Luis Norberto López de Lacalle, Francisco Fernández-Zacarías

**Affiliations:** 1 Mechanical Engineering and Industrial Design Department of School of Engineering of Puerto Real, University of Cádiz UCA. Avda. de la Universidad de Cádiz, n° 10, 11519 Puerto Real, Cádiz, Spain; francisco.fernandez@uca.es; 2 Mechanical Engineering Department of the Bilbao School of Engineers, University of the Basque Country UPV/EHU, Alameda de Urquijo s/n, 48013 Bilbao, Spain; norberto.lzlacalle@ehu.eus

**Keywords:** dry drilling, CFRP, roughness, monitoring, electric power, thrust force, torque

## Abstract

Machining parameters affects the final quality of components made in carbon fiber reinforced plastic (CFRP) composite materials. In this framework, the work here presented aims at studying the right combination of cutting speed (*v_c_*) and feed rate (*v_f_*), for dry drilling of carbon fiber reinforced plastic composite materials, which obtained better results regarding roughness, hole cylindricity, and diameter. A series of experimental tests were carried out under different drilling conditions (*v_c_/v_f_*), monitoring the thrust force (*Fz*), torque (*T*), and electric power (*EP*), to define which one can help more for industrial daily life production. Results validation was carried out using the analysis of variance, in order to relate main machining parameters cutting speed and linear feed, with thrust force, drilling torque, main spindle electric power and hole quality parameters (average roughness, cylindricity and diameter). The conclusions show that thrust force is not proportional to the cutting speed and the best combinations of cutting speed and feed were found out around the average values of tested parameters. Spindle electric power is an interesting element to take into account because it is easy to consider in real production.

## 1. Introduction

Due to their excellent properties, carbon fiber reinforced plastic (CFRP) composite materials are widely used in strategic sectors such as aeronautics, aerospace, windmill, naval and automotive, among others, where weight reduction, lower consumption and lower impact on environmental impact play important roles [1,2,3]. Thus, Davim et al. [2] studied delamination and other defects in relation to process parameters, concluding that composite damage is bigger for higher cutting speed and for higher feed.

**Figure 1:**
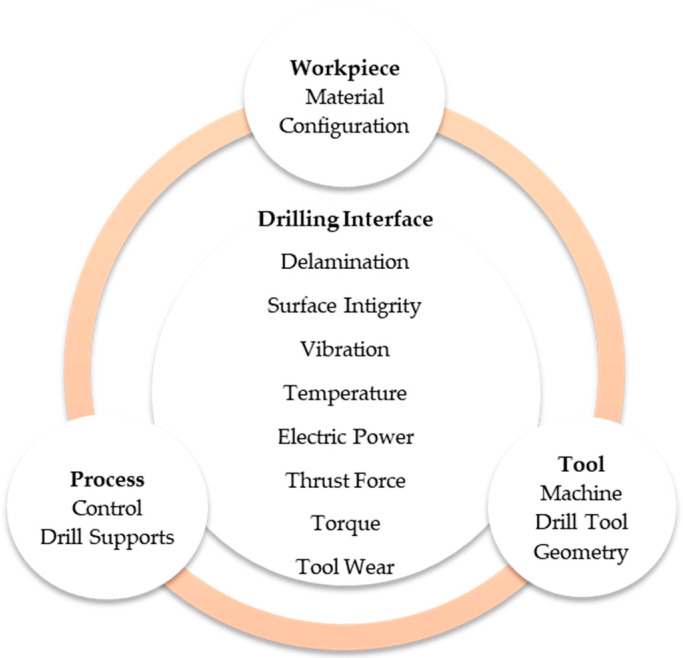
Elements involved in machining process.

In the aeronautical sector, the assembly of different aircraft parts is carried out by means of mechanical unions, such as screws, bolts and rivets, where previously, thousands or millions of holes are required [4,5,6]. Holes bad quality represents 60% of the rejects produced in CFRP parts and this generates high costs and a waste of money and time [6]. Hence, Aamir, et al. [6] gave a good classification of aspects related to hole drilling quality in CFRP, similar to Figure 1. Thus, in the Bay of Cadiz (Spain), CFRP drilling is very common due to the location of several AIRBUS facilities.

During drilling process, operation result depends on several variables [7,8], shown in Figure 1, such as (a) material to be machined (structure and stacking), (b) machining parameters (cutting speed and feed rate), and (c) drill bit characteristics (material, geometry, diameter, type of coating, main angles, number and angle of the helix, type and angle of tip and length of the transverse cutting edge) [6,9,10,11,12,13,14]. Feito et al. [9] aimed at the relation between drill point angle and thrust force when it was combined with the effect of wear progression, new tools showed negligible influence of the drill point angle on thrust force. On the other hand, one bad hole can imply the disregard of very expensive components with previous high-added value.

During drilling, inter-laminar carbon fibers and layers are brought under different compression, traction, shear or bending stresses caused by the drilling thrust force “*Fz*” and torque “*T*”, but it is affected by the orientation of carbon fibers and by cutting tool geometry (angle and cutting edge) [7,15,16]. This can occur because the fibers are alternatively subjected to torsional and compressive loads, which can cause shape defects in “cylindricity” and deviations from the nominal diameter. In this sense, some authors indicate that the most relevant defects occurring in CFRP dry drilling appears just at the drill bit input and output times; diameter deviation [17] can suffer errors of 0.23%, which may be considered unacceptable depending on the applications [18].

Ahmad et al. [18] the performance of coated tool was found to be better than its uncoated counterparts. However, despite coated WC-Co twist drills give good results and have a longer life cycle than uncoated twist drills, the latter are usually the most used in the CFRP material drilling process mainly due to their performance and good quality/price ratio. Likewise, twist drills enhanced with a double point angle generally produce less thrust force and less delamination than conventional twist drills [6,16]. Li et al. [19] carried out a comparative study using uncoated WC twist drills, one conventional and the other with a double point angle, and concluded that the latter offers less thrust force, less wear, and therefore, better quality of the holes obtained.

On the other hand, progressive tool wear affects geometry and dimensional tolerances, which can lead to defective joints, affecting the mechanical properties, reducing the load capacity and the useful life of joints, showing longitudinal cracks called splittings as the main visible fatigue damage [20]. Each particular composite can behave differently during the process, even using same tools and cutting parameters.

Monitoring of both the thrust force and torque during drilling was the subject of many investigations, intending to obtain behavioral models based on drilling parameters *v_c_* and *v_f_.* In any case, these models are only adapted to particular cases and cannot be generalized for many drill bits and all CFRP materials. However, everyone agrees that the thrust force increases with feed rate [21,22,23,24]. In this sense, Khanna et al. [25] checked that thrust force decreases at a combination of the high level of vc and low level of vf. On the contrary, there is no consensus on the behavior of the torque. Some authors indicate that the torque decreases with feed rate [21,26] and others indicate just the opposite [22,23,27,28].

Despite this, too low a feed rate values increase the contact time between drill bit and composite, which can cause inherent temperature problems and damages, as Rawat et al. [29], who considered the relation of temperature with surface delamination, surface roughness, hole circularity and hole diameter error. On the other hand, it was observed that fibers orientation influences process temperature, increases cutting forces and tool wear, being abrasion the primary wear mechanism, and thus having a direct impact on surface quality [30]. As for the temperature issue, Khanna et al. [25] compared of thrust force, roughness and cylindricity values in dry and cryogenic drilling processes. They proved that the roughness of the holes decreased by 14–38%, the values of the thrust force increased considerably (23–95%) and they obtained greater deviations from the cylindricity with cryogenic drilling in compared to dry drilling.

In other studies, drilling tests were carried out on UD-CFRP materials, concluding that the better inner hole surface quality corresponded to higher cutting speeds and lower feed rates [31]. Although some authors indicate that surface roughness is lower at high cutting speeds [32], however, others show that cylindricity and surface roughness errors increase with cutting speed [23,24], although the feed rate also contributes to increased surface roughness [24].

In order to analyze the drilling process parameters, it is necessary to acquire different variables on-line that can allow us to predict the quality of the holes without having to measure them. In this sense, there are numerous studies focused on thrust force monitoring [33,34,35], and others focused on analyzing torque [36,37,38], but few are focused on monitoring spindle electric power consumption as the work by Al-Suleiman et al. [39] demonstrated that the small difference between electrical and mechanical power for composite materials, compared to steel, is attributed to vibration and heat, and not due to a chip mechanism. Since the electric power monitoring systems are less invasive than those used to measure thrust force and torque, it makes it very interesting for industrial workshops.

In short, the purpose of using CFRP materials in the aeronautical sector is to lighten weight, and improve efficiency and safety. In this sense, reducing hole damages is key for safety and cost reduction, improving process efficiency. Taking these needs into account, this work identifies the drilling parameters combination of cutting speeds between 85 and 145 m/min and feed rates between 250 and 400 mm/min for the dry drilling of CFRP. Process criteria were the best roughness, cylindricity, and hole diameter. Likewise, the conditions of efficiency will be analyzed against the quality parameters mentioned above.

For this purpose, dry CFRP drilling tests were carried out, using different *v_c_/v_f_* cutting ratios. Thrust force, torque and spindle electric power are monitored, to know how these are affected by *v_c_* and *v_f_*, as well as its influence on the micro and macro-geometric quality of the holes obtained.

## 2. Materials and Methods

Test pieces used for the tests are CFRP sheets used in the aeronautical sector, 210 mm × 210 mm and 4.5 mm thick. It is composed of unidirectional carbon fiber layers, of intermediate modulus, with the following stacking sequence [0/90/45/−45/45/−45]. The matrix is impregnated with epoxy resin and has 34% volume before curing. The CFRP laminate was manufactured by manual laying, vacuum bag molding, and autoclave cure for 180 min at 185 °C and subjected to a pressure of 7 bar.

Regarding drilling tests, two machines were used in the tests:

- A Machining Center, Kondia (Elgoibar, Spain) Five 400 5-axis, equipped with a Heidenhain iTNC530 control.
- A High Speed Machining Center, Kondia HS 1000 3-axis, equipped with a Heidenhain iTNC530 control.

Both machines behaved similarly, with electro integrated spindles with ceramic balls. Both were very stiff and stable, with *Z*-axis stiffness over 70 N/μm.

### 2.1. Monitoring Equipment

**Figure 2:**
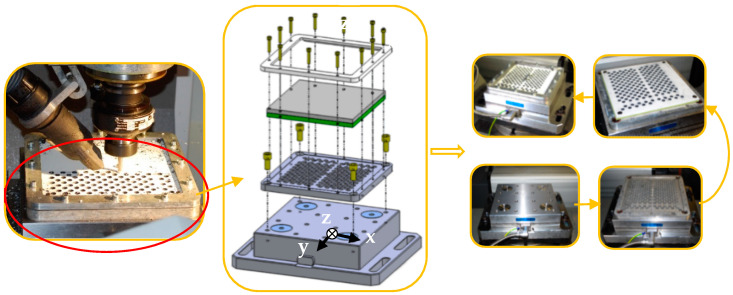
Dynamometric table and workpiece clamping system, showing the backplate support.

Kistler^®^ (Winterthur, Switzerland) model 9255B dynamometer was used for measuring the three orthogonal force component, that is, for the acquisition of thrust force and torque. This dynamometer has high rigidity and consequently, a high first resonant frequency, in the range of 2 KHz, far from drilling for excitation. Its high resolution enables the smallest dynamic changes in large forces to be measured, with a sensitivity of −3.7 pC/N. This model was used in the two machining centers, Kondia Five 400 and Kondia HS 1000. As load amplifiers, models 5019 B and 5017 B were used, both from the Kistler. The main application field of these amplifiers is the cutting force measurement with a range of sensitivity from 0.01 to 9990 pC/M.U. and bandwidth from 0–200 kHz. A special workholding backplate was installed on the dynamometer for workpiece clamping system, see Figure 2.

The data acquisition used were, for the Kondia HS 1000 machine, equipment real-time multi-analyzer and recorder Oros OR35, with the NVGate data processing software; and for the Kondia Five 400 machine, the CompactRIO equipment, with the NI9215 data acquisition card from National Instrument^®^ (Austin, TX, USA) and the specific LabView^®^ software (Labview 2015 SP1, National Instrument, Austin, TX, USA). Both data loggers allow a sampling rate of up to 100 kS/s.

Likewise, in the Kondia HS 1000 machine, the second stage in experiments, thrust force and torque were also monitored using a rotary non-contact dynamometer, type ARTIS^®^ DDU-4 (Marposs, Bentivoglio, Italy), and a spindle electric power acquisition system, model UPC, with sampling capacity up to 3 kS/s, of the brand Load Controls Software Incorporated^®^ (UPC-230, Sturbridge, MA, USA) and Matlab^®^ (V- R2019a. Natick, MA, USA) as a data acquisition system.

**Figure 3:**
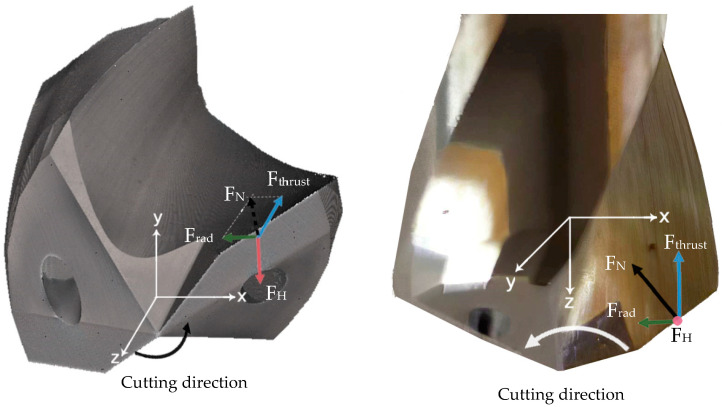
The forces exerted on a main cutting lip of the drill bit.

Figure 3 shows the forces exerted on a main cutting lip and the torque direction. The plane XY on which the horizontal force (F_H_) lies is perpendicular to the axis of the drill bit. This force generates a resistant torque because it acts itself out at a certain distance from this axis. The normal force (F_N_) can be broken down into two components, one of them perpendicular (F_rad_) and the other one parallel (F_trust_) to the Z axis.

### 2.2. Equipment Used to Check Hole Quality

The measurement systems for evaluating the quality of the holes were:

- To measure roughness (*Ra*): The roughness parameter used to assess the surface quality of the holes is the arithmetic mean roughness (*Ra*), the industrially most widely used parameter. Mahr Perthometer PGK 120 roughness tester (Göttingen, Germany), equipped with an MFW 250 probe and a selectable measurement range of ±25 µm or ±250 µm, was used.
- To measure diameters: an inside three-contact micrometer, a Mitutoyo^®^ (Kawasaki, Japan) Digimatic, with a measurement range of 6–8 mm, with a precision of 0.001 mm, and measurement uncertainty of 2 µm, was used.

To measure cylindricity: Mahr^®^ Formtester MMQ 44 machine (Göttingen, Germany) and specific FORM-PC software was used. Technical data: Roundness deviation (μm + µm/mm measuring height): 0.02 + 0.0005. This equipment has 3 linear axes X, Y, and Z and one axis of rotation, axis C, which is responsible for turning the spindle.

### 2.3. Cutting Tools: Drill Bits

Cutting tools for testing were uncoated WC-Co twist drills, SANDVIK^®^ VUS85CS0377 (Stockholm, Sweden), WC micrograin size with approx. 10% Cobalt, content see Table 1, with diameter: D = 7.92 mm, double point angle: 140°/118° and helix angle: 29.82°.

### 2.4. Experimental Methodology

On the one hand, in the high-speed machining center, Kondia Five 400, cycles of 25 consecutive drills were carried out with the same drill bit. This operation was repeated for each of the combinations of cutting speeds *v_c_* [m/min] (85, 105, 125, 145) and feed rate *v_f_* [mm/min] (250, 300 and 400), Table 2, using a new drill bit for each combination (25 holes), in total 300 holes (12 sets of 25 holes).

On the other hand, in the high-speed machining center, Kondia HS 1000, samples of 17 consecutive holes were made, for the same combinations of *v_c_* and *v_f_* previously made, using a new drill for each combination (17 holes), in total 204 holes (12 rounds of 17 holes).

Throughout the drilling process, thrust force, torque and spindle electric power were recorded, using a sampling frequency of 1 kS/s. The quality of the holes obtained are measured and evaluated, after the end of the drilling process, following a methodology according to the parameter to be measured, that is to say:

- Roughness (average roughness, *Ra*) (micro-geometric deviations): the average value of the measurements in 4 generatrices arranged at 90°.
- Diameters (macro-geometric deviations): the average of three values.
- Cylindricity (deviations of form): the MMQ 44 unit and the FORM-PC software are used.

The correct functioning of the measurement equipment was verified at the beginning of the tests, by calibrated standards. Likewise, the appropriate sampling frequencies were established based on the variables to be measured and the necessary filters depending on the type of parameter, mainly to eliminate possible high-frequency interference.

The analysis of variance for both linear and non-linear models and the multiple determination coefficient were used for the results analysis at 95% confidence level, in order to demonstrate the percentage of variance justified by the variables of the machining programming parameters. All data were analyzed using Matlab software.

## 3. Results and Discussion

Once the data acquired by the on-line monitoring systems, of thrust force (*Fz*), torque (*T*) and electric power (*EP*) have been processed and analyzed, and the off-line values of roughness (*Ra*), diameter measured (*D*) and cylindricity (*C*), taken as indicators of hole quality, we proceed to describe the results of the experimental tests. The objective is to determine the relationships between the on-line values and the off-line indicators, depending on the machining parameters used in the tests.

### 3.1. Experimental Relationship of Thrust Force, Torque and Electric Power, with Machining Parameters

**Figure 4:**
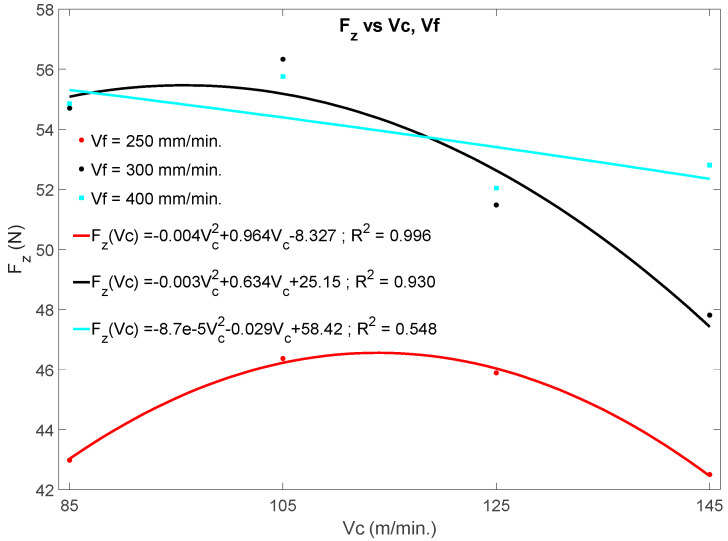
Effects of cutting speed and feed rate on the average thrust force (*Fz*).

Figure 4 shows the influence of the machining parameters, cutting speed (*v_c_*) and feed rate (*v_f_*) on the variation of the thrust force during the drilling process, as well as the empirical relationships of the thrust force with the drilling parameters. The mathematical models for the thrust force as a function of feed rates and spindle speeds, *Fz* (*v_c_*, *v_f_*), in general, are significant, except for the behavior of the representation for *v_f_* = 400 mm/min, whose coefficient of determination is around 55%.

The graphs evidence that if *v_f_* is kept constant, *Fz* shows a tendency to decrease with *v_c_*, at least for the first 25 drills and the range of cut speeds tested. It is also evident that in all cases, a maximum appears around *v_c_* = 105 m/min, which represents an indicator to compare with the quality of the holes later. On the other hand, if *v_c_* is kept constant, *Fz* tends to increase with *v_f_* and with the number of holes (*N*) [30], in relation to the higher chip section and tool wear.

**Figure 5:**
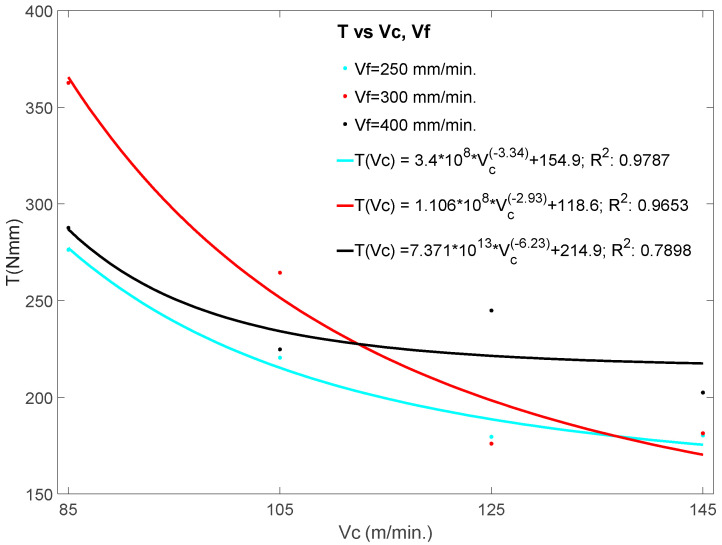
Effects of cutting speed and feed rate on the torque (*T*).

Figure 5, shows the trend of the average torque recorded during the drilling process, as a function of cutting speeds and feed rates, as well as the empirical relationships of the torque with the drilling parameters. The mathematical models for the torque as a function of feed rate and spindle speed are significant. In any case, tests demonstrated that the torque decreases with the increase in *v_c_*, however, in the case of the influence with *v_f_*, the situation cannot be generalized due to the large dispersion with a determination index below 40%. Even so, if instead of using the parameter *v_f_*, we use the parameter *f* [mm/rev], the coefficient of determination approaches 70% and, in this case, an upward trend of the torque would be detected as the parameter f increases [32]. However, in order to consolidate any conclusion with the latter case, it would be advisable to increase the number of drilling tests, taking into account the parameters “*f*” in the design of the experiment.

**Figure 6:**
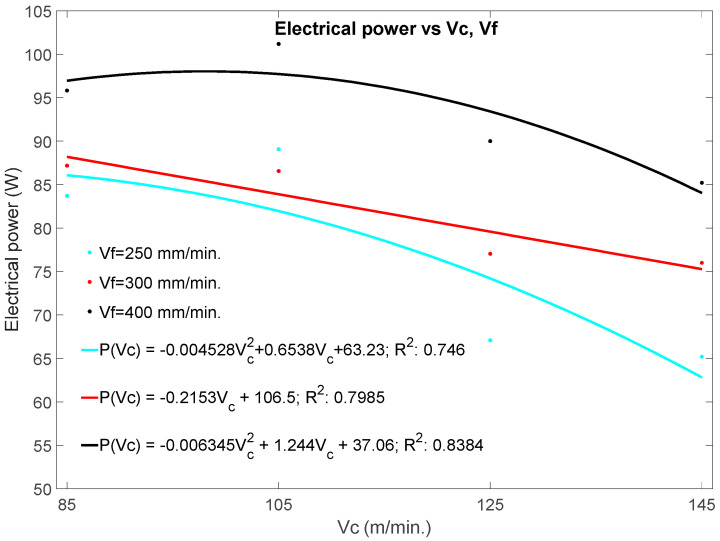
Effects of cutting speed and feed rate on the electric power (*EP*).

Figure 6 shows the electric power at the spindle as a function of the machining parameters, *v_c_*, *v_f_*, as well as the empirical relationships of the power with the drilling parameters. The graph demonstrates that the electric power decreases with the increase in the cutting speed (*v_c_*), that is, with the spindle rotation speed. As shown in Figure 4 and Figure 5, the thrust forces and torque also decrease with increasing *v_c_.* Mechanical power is proportional to speed, torque and force, but in the case of machining of composite materials, other factors that can condition these values must be taken into account, such as electric power, efficiency, and temperature.

In composite materials, the mechanical torque and thrust force can decrease with increasing cutting speed [21] and, therefore, mechanical power. This will allow to take advantage of higher values of cutting speeds, which together with a good relation of feed rates, will reduce the times and costs of the process, increasing its performance. On the other hand, the consumption of electric power can be correlated with the wear of the flank of the cutting tool and, therefore, with the quality of the holes obtained [39].

Figure 4 and Figure 6 shows a maximum from which the graphed parameters (*Fz*, *T*) decrease with the cutting speed. However, it is also appreciated that below this maximum the graphed parameters tends to increase with the cutting speed. This phenomenon could be due to an alteration in the characteristics of the CFRP material.

### 3.2. Empirical Relationship of the Thrust Force with the Number of Holes, the Feed Rate and the Cutting Speed

Previously, it has been verified that the thrust force varies depending on the number of holes, the cutting speed and the feed rate, the latter being the most significant. A behavioral model of the thrust force, *Fz = f* (*N*, *v_c_*, *v_f_*), has been obtained using the following empirical relationship (1):

*F_z_* (*N*, *V_c_*, *V_f_*) = β_0_ + β_1_*N + β_2_*V_c_ + β_3_*V_f_ + β_4_*N^2^ + β_5_*V^2^_c_ + β_6_*V^2^_f_ (1)

where *Fz* is the thrust force in Newton, *v_c_* is the cutting speed in m/min, *v_f_* is the feed rate in mm/min and *N* the number of holes. This empirical relationship with the drilling parameters is analyzed and justified in Table 3, where the results of the analysis of variance for the polynomial function of the *Fz* model are shown.

As can be seen, all *p*-Value of the regression models were significantly lower than the statistical *p*-Value of 5% at 95% of the confidence level. In addition, the linear and non-linear terms are also shown, both of them would also represent a good approximation of the behavior of *Fz*. However, the determination coefficients in these last two cases come out somewhat less than 0.95.

On the other hand, taking into account that the feed [mm/rev] is related to the cutting speed [m/min] and the feed rate [mm/min], *f = g*(*v_c_*, *v_f_*), it is possible to establish a final relationship that allows the equation to be represented in three 3 dimensions. The following model (2) is proposed for this:

*F_z_* (*N*, *f*) = β_0_ + β_1_*N + β_2_*f + β_3_*N^2^ + β_4_*f^2^ (2)

This empirical relationship of de thrust force with the drilling parameter was studied using multiple regression analysis by considering all of the experimental conditions, Table 4. All the coefficients, except the independent term, have a *p*-Value lower than 5% at 95% confidence level. The determination coefficient is close to 0.9, which is indicative of the excellent fit of the proposed model. The models proposed by Equations (1) and (2) were verified with the results obtained in the experimental tests carried out.

By focusing attention on the coefficients of Equation (1) for *v_c_* and *v_f_*, the coefficients of *v*_c_ are negative, indicating that *Fz* decreases with *v*_c_; conversely, the coefficients of *v_f_* are positive, showing that *Fz* increases with *v_f_*. This behavior can be verified in Figure 4.

**Figure 7:**
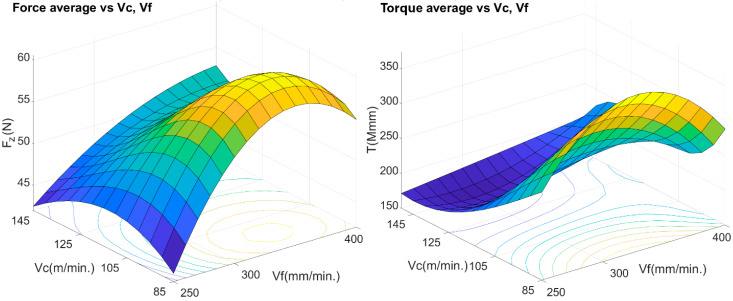
Effects of cutting speeds and feed rates on the average thrust force (*Fz*) and torque (*T*).

Figure 7 shows the data estimated from the average values of thrust forces and torque as a function of the *v_f_* and the *v*_c_. It can be seen that the functions and curves described above agree with the represented values. The graph shows that the lowest value of the average thrust force is given for cutting speeds of 85 and 145 m/min and feed rate of 250 mm/min. In the case of torques, the smallest values are given around the cutting speeds of 125 and 145 m/min and feed rates of 250 and 300 mm/min. In both cases, the highest values are given for the highest feed rates 300–400 mm/min.

### 3.3. Empirical Relationship of Roughness, Cylindricity and Diameter with Drilling Parameters

In order to determine *Fz* values and torques related to the best quality indices, i.e., roughness (*Ra*), cylindricity (c), and diameter (*D*), it is necessary to analyze these values with the results obtained in the previous sections.

**Figure 8:**
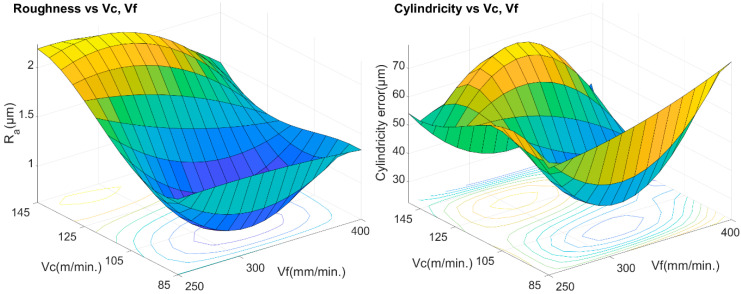
Effects of cutting speed and feed rate on the average roughness (left) and cylindricity (right).

Figure 8 shows two graphs, roughness (left) *Ra* (average) and cylindricity (right), both as a function of cutting speeds and feed rates. When comparing these values with those obtained in Figure 6, it is clearly observed that the lowest roughness values do not correspond to the lowest values of the thrust force and, for the machining parameters used in the test, a direct correlation is not observed with vf, as some authors maintain [34], for other machining parameters and other types of fiber.

The smallest roughness values are given for the experimental values of *v_c_* = 105 m/min and *v_f_* = 300 mm/min, just where the recording of average thrust forces was highest. However, in the case of cylindricity, the best results are given for the smallest *v_c_* 85–105 m/min and intermediate *v_f_* = 300 mm/min, precisely where the highest values of torques are given, for *v_c_* = 85 m/min and *v_f_* = 300 mm/min.

It was not possible to find a simple, sufficiently significant empirical relationship, with a determination coefficient greater than 40%, for roughness depending on the parameters: thrust force, torque and electric power, *R_a_ = f* (*Fz*, *T*, *EP*). However, it has been possible to determine a simple relationship as a function of the machining parameters, “*f ≈ v_f/_v_c_*”, with a determination coefficient higher than 70%. The empirical relationship is shown in Figure 8 (left), and the statistical data for a level of 95% confidence are shown in Table 5.

**Figure 9:**
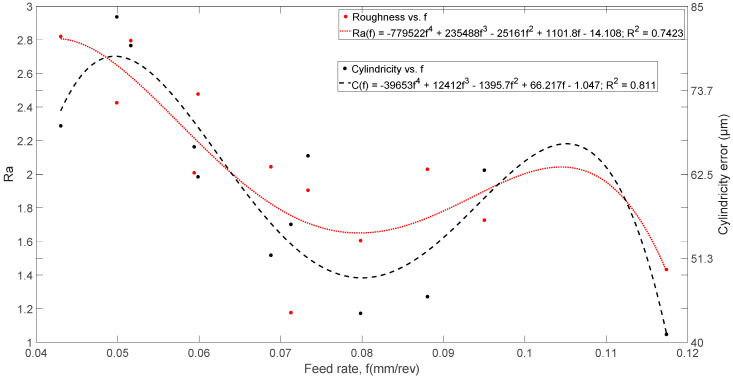
Effects of feed rates [mm/rev] on average roughness (*Ra*) and cylindricity.

Figure 9 shows roughness (*Ra*) and cylindricity trends as a function of *f* [mm/rev]. It can be seen that the trends and shapes of both are very similar, with maximum and minimum values for approximately the same *f* values. It is also verified that the lowest values are recorded in the position *v_c_* = 105 m/min and *v_f_* = 300 mm/min, results that agree with those obtained in Figure 7.

Regarding roughness (*Ra*), and with the same constraints, it was also not possible to find a significant empirical relationship with a coefficient of determination above 40% between the cylindricity and the parameters: thrust force, torque, electric power, cutting speeds and feed rates. However, it was also possible to deduce a simple relationship, with a determination coefficient higher than 70%, as a function of the feed rate “*f* [mm/rev].” The empirical relationship is shown in Figure 8 (right), and the statistical data for a confidence level of 95% are shown in Table 6. Although the coefficient of determination is less than 90%, it is not a poor indicator, as it explains more than 80% of the cylindricity data.

**Figure 10:**
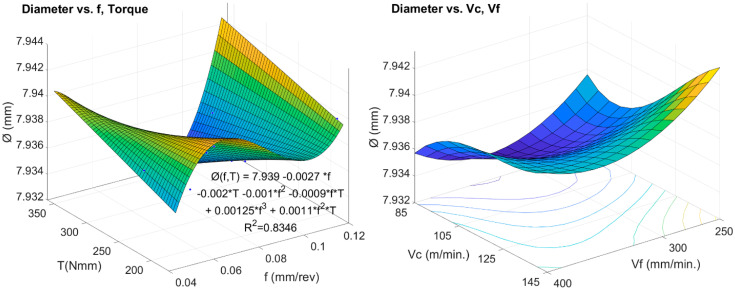
Effects of torque, cutting speeds and feed rates on the diameter of the holes.

Figure 10 shows the behavior of the recorded hole diameters as a function of *v_c_* and *v_f_* (on the right), and as a function of feed rate and torque (on the left). The lowest diameter deviations are obtained for the cutting speed of 85 m/min, the lowest *v_c_* value used in the test, and the feed rate around 300 mm/min. In the graph on the right, it can be seen that not only does *v_c_* and *v_f_* influence the diameter, but so does the torque (*T*). In this sense, it has also been verified that the thrust force (*Fz*) is influential in the quality results derived from the diameter of the holes.

The empirical relationship of the diameter with force, torque, cutting speed, and feed rate, is given by Expression (3):

*Ø* (*Fz*, *T*, *f*) = 7.976 − 0.328 *f + 0.00016 *T -0.0013229*F_z_ − 0.0026076 *T *f + 0.016794 *F_z_ *f (3)

Table 7 shows the results of the analysis of variance from multiple regression that demonstrates the goodness of fit of this expression with a *p*-Value of less than 5% at 95% confidence level and a coefficient of determination greater than 90%. This result implies that the mathematical model is statistically significant and sound.

**Figure 11:**
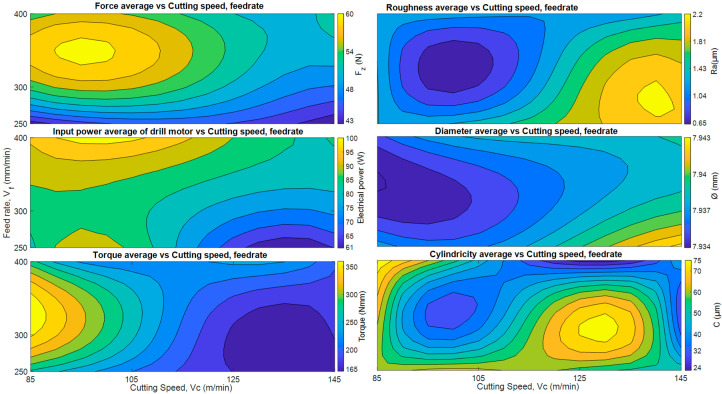
Average thrust force, torque, electric power, roughness, cylindricity and diameter.

In order to compare all the data discussed previously, in Figure 11 average values of all the parameters analyzed, thrust forces, torques, electric powers and quality factors (roughness, cylindricity and diameter) are shown, as a function of drilling parameters. Color codes have been used to represent amplitudes.

The best average values of -roughness, cylindricity and diameter-, are given in the area around *v*_c_ = 105 m/min and *v_f_* = 300 mm/min, corresponding to *f* = 0.07 mm/rev. However, the lowest values of thrust force, torque and energy consumption, occur in the area between *v_c_* 125–145 m/min and *v_f_* 250–300 mm/min, and it is interesting to note that the high values of thrust force correspond the lower values of roughness, diameter and cylindricity.

With the analysis of all these data, it is possible to determine which parameters provide the best quality indices during the drilling process for the materials studied and the cutting tool used.

In summary, and using Figure 11 as reference, taking into account the quality indices that the area that brings together the worst quality values corresponds to the high cutting speed, 145 m/min and low feed rate, 250 mm/min. On the contrary, the area where the best quality values are registered corresponds to cutting speed 105 m/min and feed rate of 300 mm/min.

However, analyzing the values of thrust force, torque, and electric power, the lowest values were obtained for the high range of *v_c_* 125–145 m/min and the ratio of *v_f_* 250–300 mm/min. This phenomenon could be largely influenced by temperature, in addition to other factors discussed above, such as the CFRP material and the tool. In this sense, thermal softening due to generated by drilling process heat was studied by Jinyang Xu et al. [40], confirming that the main source of heat generation when drilling CFRP is the friction at the tool-workpiece interaction zone, which contributes a percentage of around 71.6% to the overall drilling temperatures. They propose lower cutting speeds to reduce the temperature of the flank tool-work surface.

Thermal softening of the material could justify why the highest quality deviations were obtained while coinciding with the lowest power, torque and thrust force records, precisely in the section with the highest cutting speed, see Figure 11.

In all the cases, quality indicators used are within the tolerances allowed in the aeronautical sector. However, to decrease process time and energy, it would be desirable to analyze larger samples with the drilling parameters (*v_c_/v_f_*) for the lower values of *Fz*, torque and power, and determine how significant the quality indices are. These results will make the restrictions in the programming of the machining parameters more flexible and will allow improving process performance.

Table 8 shows the results of the analysis of variance, focused on the cutting speed with the best quality indices, that is, *v_c_* = 105 m/min. The upper part of the table indicates that in the case of cylindricity there are no significant differences between the different drilling parameters for a significance level of 5% at a 95% confidence level. Regarding the roughness index, this is not significant between *v_f_* = 300 and 400 mm/min, for a confidence level of 95%. Using the same criteria, for the diameter they are only significant for the values of *v_f_* = 300 and 400 mm/min.

## 4. Conclusions

Although all the values obtained from quality indicators were in all the cases within the tolerances required in the aeronautical sector, no bad holes were found, thus some main ideas can be pointed out:

- The best quality values were *v_c_* = 105 m/min and *v_f_* = 300 mm/min. Roughness values of holes are considerably increased by 21–140% and cylindricity are considerably increased by 5–91% under different combinations of *v_c_* and *v_f_* when compared with *v_c_* = 105 m/min and *v_f_* = 300 mm/min. On the contrary, there were no significant variations on the behavior of diameter, which was only increased by 0.2–0.3% under different combinations of *v_c_* and *v_f_* when compared with the nominal diameter.
- It was shown that for *v_c_* = 105 m/min, roughness, cylindricity and diameter variations were not significant within the range of feed *v_f_* [mm/min] = (300, 400), which can allow greater flexibility in programming according to other restrictions such as process time and energy consumption. In workshops using semiautomatic drilling machines, cutting speed/spindle rotational speed usually is kept constant for each bit diameter, so feed rate is the common parameter to modify.
- The parameter that most influences the thrust force *Fz* was feed rate, which is coherent with the dependency of force with chip section. Thrust force are considerably decreased by 3–23% under different *v_f_* when compared with *v_f_* = 300 mm/min. Likewise, it was determined that thrust force has a maximum for values below *v_c_* = 145 m/min. An increase in cutting speed, from this maximum, seems to have a softening effect on composites, perhaps because of heating due to higher cutting speeds provoking the softening of epoxy resin and the entire fiber fabric strength reduces its toughness.
- Thrust force, torque and electric power consumption increase as feed rate increase, but decrease with increasing cutting speed. In this sense, thrust force values are increased by 1–18%, torque values are increased by 15–17% and electric power are increased by 3–12% under different *v_f_* when vc = 105 m/min. This will allow, complying with the quality indicators, work with higher cutting speeds and the use of high *v_f_/v_c_* ratios. Time and costs of the drilling process with be achieved in this way.
- The empirical model relates thrust force with the number of holes, cutting speed and feed rate, especially considering that the determination coefficient has been approximately 1.
- Based on the above, electric power monitoring systems could be a non-invasive and more efficient alternative to invasive force and torque monitoring systems to implement in machining equipment as predictive systems for hole quality. Some authors define spindle monitoring as sensorless [41] and today it is a step in the concepts of machine/process surveillance [42] in 4.0 factories.

Further research is now aimed in two directions, firstly to check the effect of cantilever conditions and part stiffness in hole quality and tool wear. To do that, some cantilever-type setups are being tested. The second research line will be to implement power monitoring devices for manual semiautomatic drilling units as manual tools are the most common in aeronautical workshops.

## Figures and Tables

**Table 1:** Cutting tool geometrical parameters (VUS85CS0377).

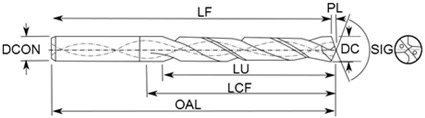						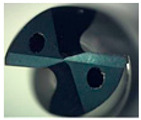	
**DC**	**LU**	**DCON**	**SIG**	**PL**	**OAL**	**LF**	**LCF**
mm	mm	mm	angle °	mm	mm	mm	mm
7.92	25.00	8.00	140/118	1.20	80.00	78.80	30.00

**Table 2:** Drilling parameters.

Parameters	Values
Drill bit diameter, φ, D (mm)	7.92
Cutting speed (m/min)	85, 105, 125, 145
Spindle speed (rpm)	3416, 4220, 5024, 5828
Feed rate (mm/min)	250, 300, 400
Feed rate (mm/rev)	0.04–0.12
Lubrication	Dry
CFRP material thickness (mm)	4.50

**Table 3:** Results of regression.

**Coef.**		**Estimate**		**SE**		**t-Stat**	***p*-Value**
β_0_		0		0		NaN	NaN
β_1_		2.1054		0.075386		27.929	8.9938 × 10^−60^
β_2_		−1.448		0.10452		−13.853	3.2856 × 10^−28^
β_3_		0.72588		0.037136		19.546	3.1831 × 10^−42^
β_4_		−0.029541		0.0028146		−10.496	1.836 × 10^−19^
β_5_		0.0056399		0.00046508		12.127	1.0158 × 10^−23^
β_6_		−0.0010371		5.6422 × 10^−5^		−18.38	1.7474 × 10^−39^
**Force Model Summary**							
N	R		**R^2^**		**Adjusted R^2^**	**Root Mean Squared Error**	
60	0.989		**0.978**		**0.978**	**1.6**	
**ANOVA**							
			**DF**	**SS**	**MS**	**F**	***p*-Value**
Total			149	16,955	113.79		
Model			5	16,587	3317.4	1296.8	8.3474 × 10^−118^
Linear			3	15,441	5147	2012	2.6839 × 10^−117^
Nonlinear			2	1146	573.01	224	6.2348 × 10^−45^
Residual			144	368.36	2.5581		

**Table 4:** Results of regression.

**Coef.**	**Estimate**		**SE**	**tStat**	***p*-Value**
β_0_	4.4132		2.5743	1.7144	0.087513
β_1_	1.9697		0.1171	16.82	5.5414 × 10^−45^
β_2_	562.76		66.356	8.4809	1.0917 × 10^−15^
β_3_	−0.026491		0.0043721	−6.0592	4.1665 × 10^−9^
β_4_	−2551.3		417.36	−6.1128	3.0938 × 10^−9^
**Force Model Summary**					
**N**	**R**	**R^2^**	**Adjusted R^2^**	**Root Mean Squared Error**	
60	0.944	0.891	0.889	3.51	
**ANOVA**					
	**DF**	**SS**	**MS**	**F**	***p*-Value**
Total	299	33,403	111.72		
Model	4	29,761	7440.2	602.66	1.447 × 10^−140^
Linear	2	28,846	14,423	1168.3	6.5596 × 10^−141^
Nonlinear	2	914.56	457.28	37.04	4.4506 × 10^−15^
Residual	295	3641.9	12.346		

**Table 5:** Roughness Model Summary.

**N**	**R**	**R^2^**	**Adjusted R^2^**	**Root Mean Squared Error**	
12	0.861	0.7423	0.595	0.329	
**ANOVA**					
	**DF**	**SS**	**MS**	**F**	***p*-Value**
Total	11	2.9437	0.26761		
Model	4	2.185	0.54626	5.0401	0.03127
Residual	7	0.75868	0.10838		

**Table 6:** Cylindricity Model Summary.

**N**	**R**	**R^2^**	**Adjusted R^2^**	**Root Mean Squared Error**	
12	0.900	0.811	0.703	0.00736	
**ANOVA**					
	**DF**	**SS**	**MS**	**F**	***p*-Value**
Total	11	0.002003	0.00018211		
Model	4	0.001625	0.00040613	7.5075	0.011273
Residual	7	0.000379	5.4097 × 10^−5^		

**Table 7:** Diameter Model Summary.

**N**	**R**	**R^2^**	**Adjusted R^2^**	**Root Mean Squared Error**	
12	0.958	0.918	0.85	0.000933	
**ANOVA**					
	**DF**	**SS**	**MS**	**F**	***p*-Value**
Total	11	6.382 × 10^−5^	5.802 × 10^−6^		
Model	5	5.859 × 10^−5^	1.172 × 10^−5^	13.449	0.00328
Residual	6	5.228 × 10^−6^	8.714 × 10^−7^		

**Table 8:** Results of analysis of variance for v_c_ = 105 m/min.

			**SS**	**df**	**MS**	**F**	**Sig.**
Roughness	Between Groups		3.590	2	1.795	7.033	0.003
	Within Groups		6.891	27	0.255		
	Total		10.481	29			
Diameter	Between Groups		0.000	2	0.000	53.977	0.000
	Within Groups		0.000	27	0.000		
	Total		0.001	29			
Cylindricity	Between Groups		568.993	2	284.496	0.272	0.764
	Within Groups		28,279.393	27	1047.385		
	Total		28,848.386	29			
**Multiple Comparisons**							
**Bonferroni**	**(I) Vf**	**(J) V_f_**	**MD (I-J)**	**SE**	***p*-Value < 5%**	**95% Confidence Interval**	
						**Lower Bound**	**Upper Bound**
Roughness	250	300	0.833000 *	0.225932	0.003	0.25632	1.40968
	300	250	−0.833000 *	0.225932	0.003	−1.40968	−0.25632
Diámeter	250	300	0.008700 *	0.000935	0.000	0.00631	0.01109
		400	0.008100 *	0.000935	0.000	0.00571	0.01049
	300	250	−0.008700 *	0.000935	0.000	−0.01109	−0.00631
	400	250	−0.008100 *	0.000935	0.000	−0.01049	−0.00571

* The mean difference is significant at the 0.05 level.
